# Polymer-Particle Pressure-Sensitive Paint with High Photostability

**DOI:** 10.3390/s16040550

**Published:** 2016-04-16

**Authors:** Yu Matsuda, Kenta Uchida, Yasuhiro Egami, Hiroki Yamaguchi, Tomohide Niimi

**Affiliations:** 1 Institute of Materials and Systems for Sustainability, Nagoya University, Furo-cho, Chikusa-ku, Nagoya, Aichi 464-8603, Japan; 2 Department of Micro-Nano Systems Engineering, Nagoya University, Furo-cho, Chikusa-ku, Nagoya, Aichi 464-8603, Japan; uchida.kenta@f.mbox.nagoya-u.ac.jp (K.U.); hiroki@nagoya-u.jp (H.Y.); niimi@mech.nagoya-u.ac.jp (T.N.); 3 Department of Mechanical Engineering, Aichi Institute of Technology, 1247 Yachigusa, Yakusa-cho, Toyota, Aichi 470-0392, Japan; egami@aitech.ac.jp

**Keywords:** pressure-sensitive paint, polymer particles, photostability, polymer-particle pressure-sensitive paint

## Abstract

We propose a novel fast-responding and paintable pressure-sensitive paint (PSP) based on polymer particles, *i.e.* polymer-particle (pp-)PSP. As a fast-responding PSP, polymer-ceramic (PC-)PSP is widely studied. Since PC-PSP generally consists of titanium (IV) oxide (TiO_2_) particles, a large reduction in the luminescent intensity will occur due to the photocatalytic action of TiO_2_. We propose the usage of polymer particles instead of TiO_2_ particles to prevent the reduction in the luminescent intensity. Here, we fabricate pp-PSP based on the polystyrene particle with a diameter of 1 μm, and investigate the pressure- and temperature-sensitives, the response time, and the photostability. The performances of pp-PSP are compared with those of PC-PSP, indicating the high photostability with the other characteristics comparable to PC-PSP.

## 1. Introduction

Pressure-sensitive paint (PSP) is a powerful pressure measurement technique [1,2,3,4] and has been applied to various studies [5,6,7,8]. In general, PSP consists of a dye molecule and a binder (polymer layer or porous material) [1,2]. When the PSP layer applied to a surface of interest is illuminated by a UV or blue light (~400 nm), the dye molecules are excited and emit luminescence (phosphorescence or fluorescence). The luminescence from the dye molecule used in PSP can be quenched by the interaction with oxygen molecules (oxygen quenching); thus, the pressure measurement is conducted by measuring the luminescent intensity. In recent years, unsteady flow field measurement techniques have been a major topic in the PSP researchers. There are two approaches in unsteady PSP studies: One is the development of fast-responding PSPs [3,4,9,10,11], and the other is that of measurement algorithms [12,13,14]. In general, porous binders are employed for fast-responding PSPs, because those binders have a short time-constant of the oxygen diffusion in the binder [1,4]. Anodized aluminum (AA-)PSP is known as a typical porous PSP [3,4,9,10,11], but AA-PSP can only be applied to aluminum surfaces. Moreover, it is difficult to form a uniform AA-PSP layer on a model surface of a complicated shape. Therefore, polymer-ceramic (PC-)PSP, whose binder consists of polymer and ceramic particles, has received much attention [3,4,15,16,17,18,19], because PC-PSP can be applied even to a model surface of non-aluminum and of complicated shape. It is considered that mixing ceramic particles enlarge the PSP surface area and enhance its response time to pressure change. In most PC-PSP, titanium (IV) oxide (TiO_2_) particles have been used as ceramic particles [15,19]. The mixing of TiO_2_ particles will induce the reduction in photostability of PC-PSP, since TiO_2_ works as a photocatalyst under illumination of UV light (<390 nm). Therefore, the number of runs by a single PC-PSP sample will be strictly limited in wind tunnel testing. A new fast-responding PSP, which is paintable and photostable, is required.

We propose a novel porous PSP called polymer-particle pressure-sensitive paint (pp-PSP), wherein polymer particles are adopted instead of TiO_2_ particles in PC-PSP. We prepared polystyrene (PS) polymer particles by a self-organized precipitation (SORP) method [20,21]. The basic characteristics of pp-PSP (pressure- and temperature-sensitivities, time response, and photostability) are investigated and compared with TiO_2_-based PC-PSP in this article.

## 2. Pressure- and Temperature Sensitivities of PSP

To obtain pressure distribution, the variation of the luminescent intensity of PSP is measured. The relation between the luminescent intensity and pressure can be described by the following Stern-Volmer equation [1,2],

(1) $$\frac{I_{\text{ref}}\left(p_{\text{ref}},T_{\text{ref}}\right)}{I\left(p,T\right)}=A\left(T\right)+B\left(T\right)\frac{p}{p_{\text{ref}}}$$

where I, p, and T are respectively the luminescent intensity, pressure, and temperature. The subscript, ref, indicates the reference condition. The constants A and B, having temperature dependency, are called the Stern-Volmer constants. It should be noted that the luminescent intensity is a function of temperature as well as pressure. In this study, we consider the temperature effect as:

(2) $$\frac{I\left(p,T\right)}{I_{\text{ref}}\left(p_{\text{ref}},T_{\text{ref}}\right)}=C+D\frac{T}{T_{\text{ref}}}$$

where C and D are constants. The partial derivatives of Equations (1) and (2) with respect to pressure and temperature can be expressed as follows.

(3) $$S_{p}=\frac{\partial }{\partial p}\frac{I_{\text{ref}}\left(p_{\text{ref}},T_{\text{ref}}\right)}{I\left(p,T\right)}=\frac{B}{p_{\text{ref}}}\;\left[1/\text{kPa}\right]$$

and

(4) $$S_{T}=\frac{\partial }{\partial T}\frac{I\left(p,T\right)}{I_{\text{ref}}\left(p_{\text{ref}},T_{\text{ref}}\right)}=\frac{D}{T_{\text{ref}}}\;\left[1/K\right]$$

Here, $S_{p}$ and $S_{T}$ denote the pressure- and temperature-sensitivities, respectively [15].

## 3. Materials and Methods

### 3.1. Preparation of Polymer Particles

**Figure 1:**
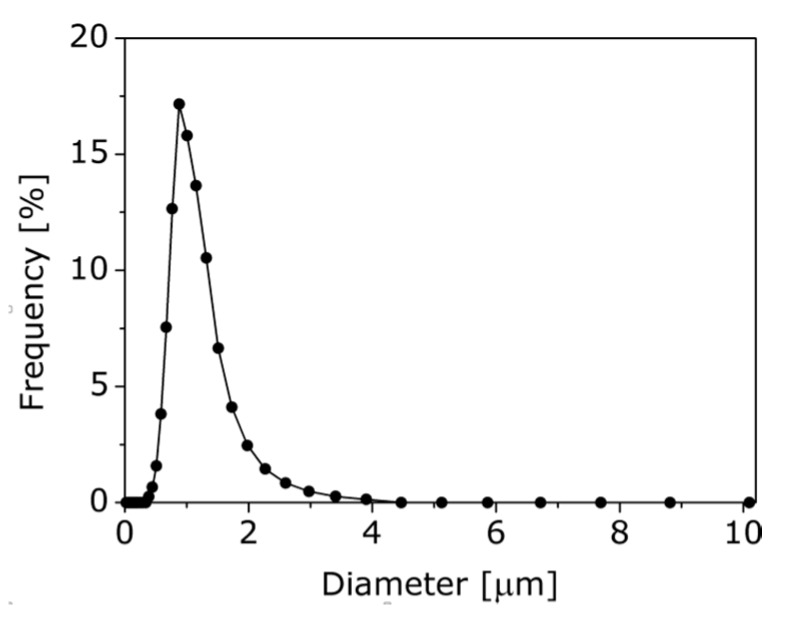
Obtained polymer particle size distribution.

We prepared polymer particles by following the self-organized precipitation (SORP) method proposed by references [20,21]. Polymer particles can be easily fabricated as follows: First, the polymer is dissolved in a good solvent. In a good solvent, the solvent favorably interacts with the polymer; that is, polymer is expanded in it. Second, a poor solvent, in which polymer is collapsed, with a higher boiling point than the good solvent, is mixed into the solution, and the obtained solution is allowed to stand at room temperature. Lastly, the polymer particles can be obtained in the poor solvent after complete evaporation of the good solvent. Polystyrene (PS: analytical grade, $M_{w}\sim 94900$, $M_{n}/M_{w}\sim 1.06$, Sigma Aldrich, St. Louis, MO, USA) was adopted in this study. Tetrahydrofuran (THF) and distilled water were respectively used as a good and poor solvent for PS. PS of 30 mg was dissolved in THF of 75 mL. Then, the distilled water of 150 mL was mixed into the PS solution. The PS particles were obtained after the evaporation of THF. The particle size distribution was analyzed by the Mie scattering analyzer (LA-920, Horiba, Kyoto, Japan) as shown in Figure 1. The arithmetic mean particle diameter and arithmetic variance were respectively 1.0 µm and 1.7 × 10^−1^ µm^2^. The polymer particles with a narrow distribution of diameters were successfully obtained. The obtained polymer particles with a diameter of 1.0 µm exhibited a white color due to light scattering.

### 3.2. Preparation of pp-PSP

We fabricated pp-PSP in three steps: First, the poly(4tBS) (poly(4-tert-butyl styrene), Sigma Aldrich, St. Louis, MO, USA) toluene solution with a concentration of 8.1 g/L was prepared. This solution of 3.0 mL was sprayed onto an aluminum plate (20 × 20 mm) as a base layer. Second, the ethanol solution of polymer particles (polymer particle: ethanol = 30 mg: 10 mL) was sprayed onto the base layer. Lastly, the ethanol solution of PtTFPP (platinum (II) meso-tetra(pentafluorophenyl) porphine, Frontier Scientific, Logan, UT, USA) with a concentration of 1.4 × 10^−2^ g/L was sprayed onto the polymer particle layer (the PtTFPP solution of 5.0 mL was sprayed). In between each step, the layer was dried for about 8 h to remove the solvents. The arithmetic average roughness of the fabricated pp-PSP surface was measured as $R_{a}=0.93\;\mu m$ by a laser microscope (VK-X200, Keyence, Osaka, Japan). This is equivalent to the diameter of the polymer particles.

We also fabricated PC-PSP to compare the photostability following the same procedure as pp-PSP except the use of TiO_2_ particles (Sigma Aldrich, St. Louis, MO, USA) instead of employing polymer particles. We prepared 5 samples each for pp-PSP and PC-PSP.

## 4. Results and Discussion

### 4.1. Pressure- and Temperature-Sensitivities of pp-PSP

**Figure 2:**
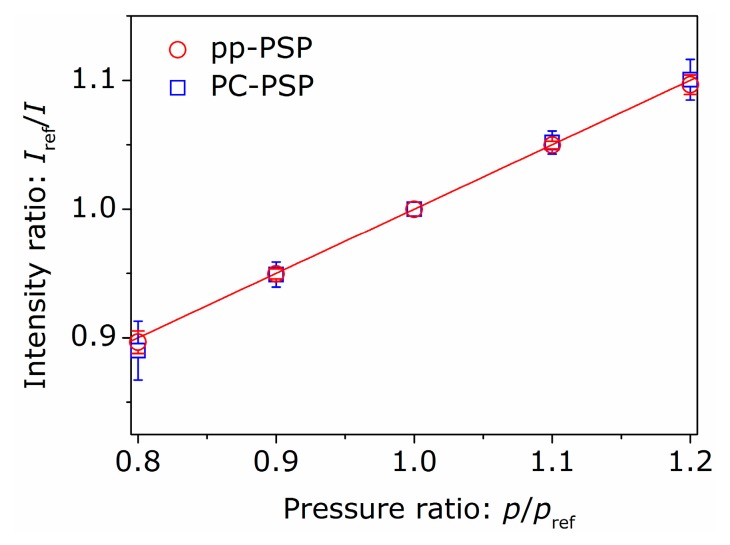
Stern-Volmer plots for polymer-particle pressure-sensitive paint (pp-PSP) and polymer-ceramic pressure-sensitive paint (PC-PSP), where $p_{\text{ref}}=100\;\text{kPa}$.

We investigated the dependencies of the luminescent intensity on pressure or temperature for the fabricated pp-PSP samples using the same experimental apparatus as our previous studies [13,22]. The wavelength of an illumination light source was 395 nm (LEDH294-395, output power: 1.4 W, Hamamatsu Photonics, Hamamatsu, Japan), and the only emission light from the sample was detected by using a band-pass filter placed in front of the camera lens. Figure 2 shows the Stern-Volmer plots for the pp-PSP and PC-PSP samples. The reference pressure was set as $p_{\text{ref}}=100\;\text{kPa}$, and $I_{\text{ref}}$ was the luminescent intensity at $p_{\text{ref}}$. The temperature of the sample was kept at $T_{\text{ref}}=293\;K$. The error bars show the standard deviation calculated from 5 independent experiments. As shown in Figure 2, the pressure dependency of pp-PSP is similar to that of PC-PSP. By fitting the Stern-Volmer plot with Equation (1) as shown in the solid line in the figure, we calculated the pressure-sensitivity defined in Equation (3). The calculated pressure-sensitivity of $S_{p}=\left(0.50\pm 0.02\right)\times 10^{-2}\;1/\text{kPa}$ was slightly lower than that of conventional polymer-based PSPs consists of PtTFPP [1]. One possible reason for this is that the dye molecules (PtTFPP) were only adsorbed on the surface of polymer-particle layer. Compared with conventional PSPs, more dye molecules will be quenched at the measured pressure range.

**Figure 3:**
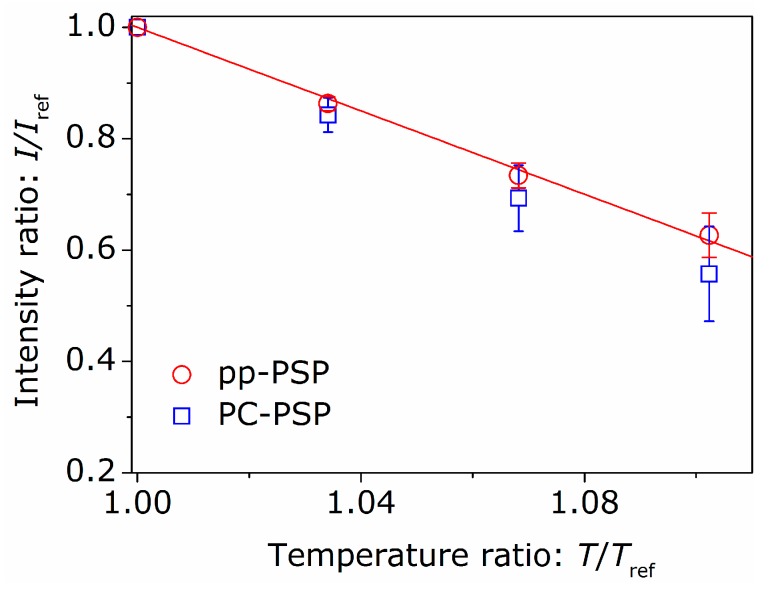
Relation between luminescent intensity and temperature for pp-PSP and PC-PSP, where $T_{\text{ref}}=293\;K$.

The temperature dependency of the pp-PSP sample was also investigated. The measurements were carried out in the pressure of $p_{\text{ref}}=100\;\text{kPa}$. The results for pp-PSP and PC-PSP are shown in Figure 3. The fitted line of Equation (2) for pp-PSP is also shown in Figure 3. Although the temperature dependency of pp-PSP is smaller than that of PC-PSP at a glance, the temperature-sensitivities calculated from Equation (4) were similar. The temperature-sensitivity of pp-PSP was calculated as $S_{T}=-\left(1.4\pm 0.1\right)\times 10^{-2}\;1/K$, and that of PC-PSP was $S_{T}=-\left(1.5\pm 0.1\right)\times 10^{-2}\;1/K$. These temperature-sensitivities are similar to that of [16], but are smaller than that of [17].

### 4.2. Time Response Property of pp-PSP

**Figure 4:**
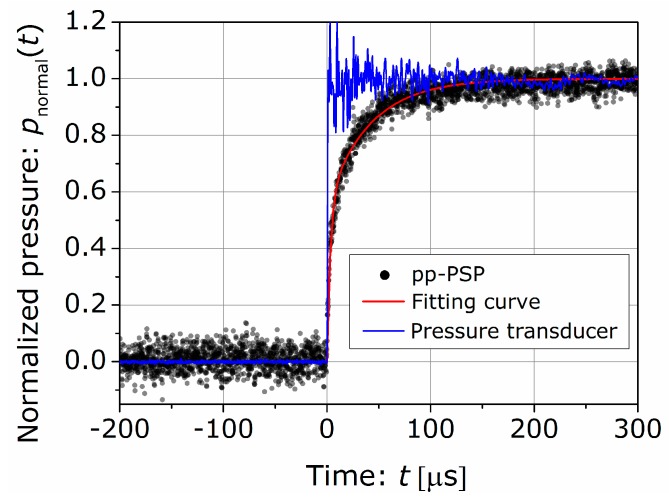
Time response test for pp-PSP.

We investigated the response time of the pp-PSP sample under a sudden stepwise pressure change by using a shock tube. The experimental setup of the shock tube was the same as our previous study [11]. The pp-PSP sample was set on the end wall of the shock tube (low pressure section) under an initial pressure of $p_{L}=20\;\text{kPa}$. The pressure of the high-pressure section was initially set at 260 kPa. Once the shock wave reached the pp-PSP sample, the sample was exposed to a sudden stepwise pressure change from $p_{L}$ to $p_{\text{impact}}$. The time-resolved pressure p(t) was calculated from the variation of the intensity of PSP measured by a photomultiplier tube. The measured pressure p(t) was normalized by the following equation [3,11,18]:

(5) $$p_{\text{normal}}\left(t\right)=\frac{p\left(t\right)-p_{L}}{p_{\text{impact}}-p_{L}}$$

where $p_{\text{normal}}\left(t\right)$ is the normalized pressure. The normalized pressure as a function of time is shown in Figure 4 as dots. The time of t=0 was defined as the shock arrival time to the end wall and was determined from the data obtained by a pressure transducer also attached to the end wall. The pressure rise time measured by the pressure transducer was less than 1.0 μs as shown in the solid blue line in Figure 4. Here, we consider the two-layered structure model [18,19] as follows:

(6) $$p_{\text{normal}}\left(t\right)=\alpha_{0}+\alpha_{1}\text{exp}\left(-\frac{t}{\tau_{1}}\right)+\alpha_{2}\text{exp}\left(-\frac{t}{\tau_{2}}\right)$$

where $\alpha_{i}$ (i=0, 1, 2) and $\tau_{i}$ (i=1, 2) are the constants. Although $\tau_{i}$ show the time constants containing the effect of lifetime of the luminescence, the diffusion coefficient, and the thickness of the layer [19], we determined them as fitting parameters in this study. Since $p_{\text{normal}}\left(t\to 0\right)\to 0$ and $p_{\text{normal}}\left(t\to \infty \right)\to 1$, Equation (6) can be expressed as

(7) $$p_{\text{normal}}\left(t\right)=1+\alpha_{1}\text{exp}\left(-\frac{t}{\tau_{1}}\right)+\left(-1-\alpha_{1}\right)\text{exp}\left(-\frac{t}{\tau_{2}}\right)$$

As shown in the solid red line in Figure 4, the experimental data with t≥0 was well fitted by Equation (7) by the nonlinear least squares fitting (the trust-region algorithm). The constants were calculated as $\alpha_{1}=-0.56\pm 0.01$, $\tau_{1}=3.3\pm 0.2\;\mu s$, and $\tau_{2}=42\pm 1\;\mu s$, where errors show the 95% confidence bounds calculated from the Student’s *t* cumulative distribution function. The response time defined as the time taken for normalized pressure to reach 0.9 was 62±1 μs. This response time was similar to that of PC-PSP [15,17,18].

### 4.3. Photostability of pp-PSP

**Figure 5:**
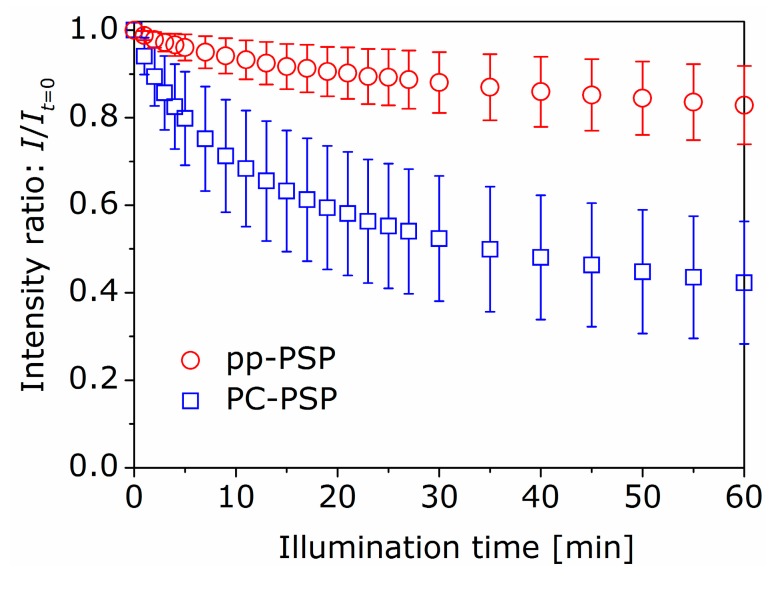
Relation between luminescent intensity and illumination time for pp-PSP and PC-PSP.

The photostability of pp-PSP was compared with that of PC-PSP. The experiment was carried out using the same apparatus as shown in Section 4.1. In this section, the distance between the LED and PSP samples was set at 40 cm to investigate the photostability under a relatively strong illumination condition. The pp-PSP and PC-PSP samples were placed side by side, and were exposed under the continuum illumination during the measurement. The luminescent intensities of both samples were measured and shown in Figure 5, where the vertical axis shows the intensity normalized by the intensity at t=0. The plots and errors shown in Figure 5 respectively show the mean and standard deviation values for the five independent experiments. The luminescent intensity of PC-PSP reduced to 42% of the initial intensity after 60 min of illumination. It is thought that this large reduction was induced by the photocatalytic action of TiO_2_. The luminescent intensity of pp-PSP reduced to 83% of the initial intensity at 60 min. This result shows that the photostability of pp-PSP is much higher than that of PC-PSP.

## 5. Conclusions

We have proposed polymer particle pressure-sensitive paint (pp-PSP). The polymer particles were adopted instead of TiO_2_ of polymer-ceramic (PC-)PSP. The polymer particles of polystyrene with narrow diameter distribution were successfully fabricated by the SORP method. The pressure- and temperature-sensitivities were similar to those of PC-PSP. The time response test showed the response time of 60 ± 1 μs for a step pressure change for pp-PSP. The reduction in the luminescent intensity was much smaller than that of PC-PSP under the continuum illumination. The high photostability was achieved in pp-PSP without degrading the pressure-sensitivity and response time of PC-PSP. Since pp-PSP with higher photostability will increase the productivity in industrial facilities, the pp-PSP is promising as a fast-responding PSP.
